# Norcantharidin Induced DU145 Cell Apoptosis through ROS-Mediated Mitochondrial Dysfunction and Energy Depletion

**DOI:** 10.1371/journal.pone.0084610

**Published:** 2013-12-19

**Authors:** Bo Shen, Pei-Jie He, Chun-Lin Shao

**Affiliations:** 1 Institute of Radiation Medicine, Fudan University, Shanghai, China; 2 Eye, Ear, Nose and Throat Hospital, Fudan University, Shanghai, China; National Institutes of Health, United States of America

## Abstract

Norcantharidin (NCTD), a demethylated analog of cantharidin derived from blister beetles, has attracted considerable attentions in recent years due to their definitely toxic properties and the noteworthy advantages in stimulating bone marrow and increasing the peripheral leukocytes. Hence, it is worth studying the anti-tumor effect of NCTD on human prostate cancer cells DU145. It was found that after the treatment of NCTD with different concentrations (25-100 μM), the cell proliferation was significantly inhibited, which led to the appearance of micronucleus (MN). Moreover, the cells could be killed in a dose-/ time-dependent manner along with the reduction of PCNA (proliferating cell nuclear antigen) expression, destruction of mitochondrial membrane potential (MMP), down-regulation of MnSOD, induction of ROS, depletion of ATP, and activation of AMPK (Adenosine 5‘-monophosphate -activated protein kinase) . In addition, a remarkable release of cytochrome *c* was found in the cells exposed to 100 μM NCTD and exogenous SOD-PEG could eliminate the generation of NCTD-induced MN. In conclusion, our studies indicated that NCTD could induce the collapse of MMP and mitochondria dysfunction. Accumulation of intercellular ROS could eventually switch on the apoptotic pathway by causing DNA damage and depleting ATP.

## Introduction

Prostate cancer (PCa) was the sixth leading cause of male cancer death in the western developed countries [1,2]. According to the recent epidemiological survey, the incidence of PCa in China is rising rapidly, especially in some developed districts [3]. Androgen-deprivation therapy (ADT) is the major strategy to inhibit the tumor growth and decrease the level of prostate-specific antigen (PSA) at the early stage. However, after about 1-3 years of treatment period, almost all tumors will eventually develop to castration-resistant PCa (CRPC) with the distant metastases and a poor prognosis [4]. Therefore, how to achieve a satisfied regression on prostate cancer under androgen-deprived conditions, still remains a challenge for the effective treatment of PCa.

Cantharidin is a natural toxin extracted from blister beetles. In ancient China, Mylabris, the dried body of the blister beetle, was used to remove furuncles, piles and warts  or to treat fistulae of tubercullous lymphadenitis. Untill Year 1810, a French chemist firstly extracted cantharidin as an active ingredient and demonstrated its definitely toxic and poisonous properties [5]. Cantharidin has been demonstrated the inhibitory effects on protein phosphatases type 2A (PP2A), which is a ubiquitous and conserved serine/threonine phosphatase with broad substrate specificity and diverse cellular functions [6,7]. Cantharidin can influence the phosphorylation of PI3K/Akt/mTOR and Mitogen-activated protein-kinase (MAPK) proteins by inhibiting the function of PP2A on activating the apoptotic pathway and regulating cell survival and proliferation [8,9]. However, the applications of cantharidin in clinic were limited because of its serious toxicity to mucous membranes in the gastrointestinal tract, urethra and kidney [10]. Norcantharidin (NCTD), a demethylated analog of cantharidin, was synthesized to reduce the toxic side-effects and retain the utility of cantharidin. It has been demonstrated that NCTD could inhibit the proliferation and induce apoptosis in several tumor cells, such as leukemic cells, gallbladder carcinoma cells, and colorectal cancer cells [11–13]. MAPK pathway, caspase-mitochondrial pathway and NF-κB pathway may be involved in the biological effect of NCTD [14–16]. In addition, due to stimulating bone marrow and increasing the peripheral leukocytes, NCTD may be a potential chemotherapeutic agent in cancer treatment [17]. 

In this study, the effects of NCTD on cell proliferation inhibition and apoptosis induction in DU145 cells were investigated. The nuclei morphological alteration, the consumption of intercellular ATP, ROS generation, and mitochondria activation-related events such as the release of cytochrome *c*, the depolarization of mitochondial membrane were respectively studied to elucidate the potential mechanism involved in NCTD-induced apoptosis of DU145 cells.

## Materials and Methods

### Cell culture

DU145 cells, an androgen-independent human prostate cancer cell line from ATCC (VA, USA), were generously provided by Dr Hongning Zhou (Columbia University, USA). Cells were maintained in the Dulbecco’s Modified Eagle medium (HyClone, Beijing, China) containing glucose (4.5 g/L) and supplemented with penicillin (100 units/ml), streptomycin (100 μg/ml), glutamate (2 mM) and 10% fetal bovine serum (Gibco Invitrogen, Grand Island, NY, USA). The culture was cultured in a humidified atmosphere of 5 % CO_2_ in air at 37°C. 

### Preparation of NCTD and treatment

NCTD was purchased form Sigma-Aldrich Chemical Company. It was dissolved in phosphate-buffered saline (PBS) to a concentration of 1M as stock solution. Serial concentrations of NCTD were diluted in culture medium before use. In this experiment, the cell culture medium was replaced with fresh medium containing various concentrations of NCTD and incubated for indicated periods from 24 h to 72 h. 

### Cell viability assay

Cell proliferation was evaluated by the methyl thiazolyl tetrazolium (MTT) assay. Briefly, DU145 cells were seeded into 96-well plates (5000 cells per well in 100 μL) and allowed to adhere overnight. After treated with indicated concentrations of NCTD for 24 h, the culture medium was replaced by 100 μL medium containing 0.5 mg/ml MTT and the plates was incubated for additional 4 h at 37°C. The medium was then removed and replaced by 100 μL of 0.04M HCl/isopropanol to dissolve the dark blue crystal thoroughly. The absorbance at 545 nm was measured by a microplate reader (Bio-Tek, USA) and cell viability was calculated by the formula: cell viability (%) = (1-OD_treatment_/OD_control_)×100 %. In another experiment, cells were treated with 25 μM NCTD for 24, 48 and 72h and for the time course.

### Flow cytometric analysis of apoptosis

Apoptosis was analyzed by using the FITC Annexin V Apoptosis Detection Kit (BD, USA) under the manufacturer’s instruction. Briefly, DU145 cells containing adherent and floating were collected by centrifugation and washed twice with cold PBS after treatment with different concentrations of NCTD for 24 h. The harvested cells were stained with FITC-conjugated Annexin-V (Annexin-V-FITC) and propidium iodide (PI) in binding buffer in a 100 μL reaction volume for 20 min in dark at room temperature. At the end of incubation, 300 μL binding buffer was added and the samples were analyzed immediately with flow cytometry. Due to PI only binding DNA in the cells having a non-intact plasma membrane, the cells stained with Annexin-V-FITC but without PI were regarded as apoptotic cells. Ten thousand events in the gate were counted for each sample. 

### Determination of mitochondria membrane potential (Δψm)

DU145 cells were harvested and stained with Rhodamine 123 to determine the mitochondria membrane potential (MMP). Rhodamine 123, a tracer dye which can enter the matrix and bind to mitochondria specifically, is widely used to detect the mitochondrial depolarization depending on the reduced fluorescence [18]. Briefly, after treatment with various concentrations of NCTD, the culture was replaced with the fresh medium containing 5μg/ml Rhodamine 123 and incubated for 20 min in dark at 37°C. Subsequently, the unbounded probes were washed out and the cells were trypsinized and harvested for flow cytometry analysis. Ten thousand events in the gate were counted for each sample.

### Nuclear morphology observation by Hoechst 33258 and TUNEL assay

Cells were seeded on lab-Tek chamber (Nunc, Thermo USA). After treatment, the slides were fixed in 4% paraformaldehyde for 15 min at room temperature. Subsequently, cells were washed triply with PBS followed by staining with Hoechst 33258 (5μg/ml, Sigma) for 10 min in dark. At the end of incubation, the cells were washed and the images were captured under a fluorescence microscopy (Olympus BX 51, Tokyo, Japan) with excitation wavelength of 330-380 nm. 

TUNEL assay (Beyotime, China) was also used to visual apoptotic cells directly. Cells were performed according to the manufacturer’s instructions. Briefly, the cultures were permeabilized with 0.1% (v/v) Triton X-100 after fixed. The slices were rinsed and covered with the TUNEL staining solution to bind the fluorescein-dUTP to the DNA break terminals. Slices were then mounted on slides for visualization through a fluorescence microscope with excitation wavelength of 450-500 nm.

### Determination of DNA fragmentation by micronucleus (MN) assay

After exposure to NCTD, the cells were fixed *in situ* with methanol/acetic acid (9:1 v/v) for 10 min. Air-dried cells were stained with 0.01 % acridine orange (Sigma-Aldrich Co.). The images were observed under a fluorescence microscope using a 40× magnification. At least 500 cells were scored and the percentage of cells with MN formation (Y_MN_) and the binucleated cells (Y_BN_) were calculated with equations: Y_MN_= A/C×100 %, Y_BN_=B/C×100 %, where A was the total number of cells with MN, B was the total number of BN cells and C was referred to the total number of cells. In some experiments, SOD-PEG (500 U/ml) was added in the medium 1 hour previous to NCTD. 

### Measurement of intracellular reactive oxygen species

The intracellular ROS level was detected using an oxidation sensitive fluorescent probe (DCFH-DA) (Beyotime, China). DCFH-DA can be deacetylated by nonspecific esterase to form DCFH that can be oxidized by hydrogen peroxide or low-molecular-weight peroxides to produce the fluorescent compound 2’,7’-dichlorofluorescein (DCF) and it is a stable fluorescent ROS-sensitive compound and can readily diffuse into cells. In this study, DU145 cells were incubated with NCTD for 24 h. The cells were harvested and washed with serum-free medium for 3 times. Aliquots of cells were re-suspended in fresh medium without serum and loaded with DCFH-DA (30 μM ) for 20 min at 37°C, according to the manufacturer’s instruction. Green fluorescence density of 10,000 events was detected by flow cytometry for each sample. 

### Measurement of mitochondrial superoxide anion

The mitochondrial production of superoxide anion (O^2^∙−) was detected with MitoSOX Red probe (Invitrogen, USA) as previously described [19]. MitoSOX Red reagent is a live-cell permeant dye and it can rapidly and selectively targete to mitochondria and be oxidized by superoxide to yield fluorescence. In our experiment, cells were incubated with MitoSOX (5 μM) in serum-free culture medium for 30 min at 37°C. Flow cytometry was used to analyze the fluorescent density. At least 10,000 events were collected from each sample.

### ATP evaluation

The cultures of an equal number of control and treated cells were scraped off with a plastic scraper and washed 3 times with the cold PBS. Whole-cell extracts were prepared by suspending pellets in lysis buffer. Intracellular ATP of lysate was measured by luciferase activity following the procedure described in the Bioluminescence Detection kit for ATP (Promega Co. USA). The values were normalized with the cell numbers.

### Western blot analysis

After treatment, cell lysate was prepared as described by He et al [20]. An equal amount of total protein were subjected to 10 % SDS-PAGE and transferred to PVDF membrane (Millipore, Bedford, USA). The following antibodies were used to probe the corresponding proteins: anti-PCNA (CST, USA), anti-cytochrome *c* (Bioworld, China), anti-MnSOD (Abcam, anti-phospho-AMPKα (Thr172, CST, USA), anti-AMPKα (CST, USA) and anti-β-actin (Beyotime, China). β-actin was used for the loading control. 

### Statistics

Experimental data were presented as the mean with standard deviation for at least three independent experiments and analyzed with the SPSS 13.0 software. The difference between groups was assessed using the Student’s *t*-test and *P*<0.05 was considered to be significant. 

## Results

### NCTD inhibited the growth of DU145 cells

**Figure 1:**
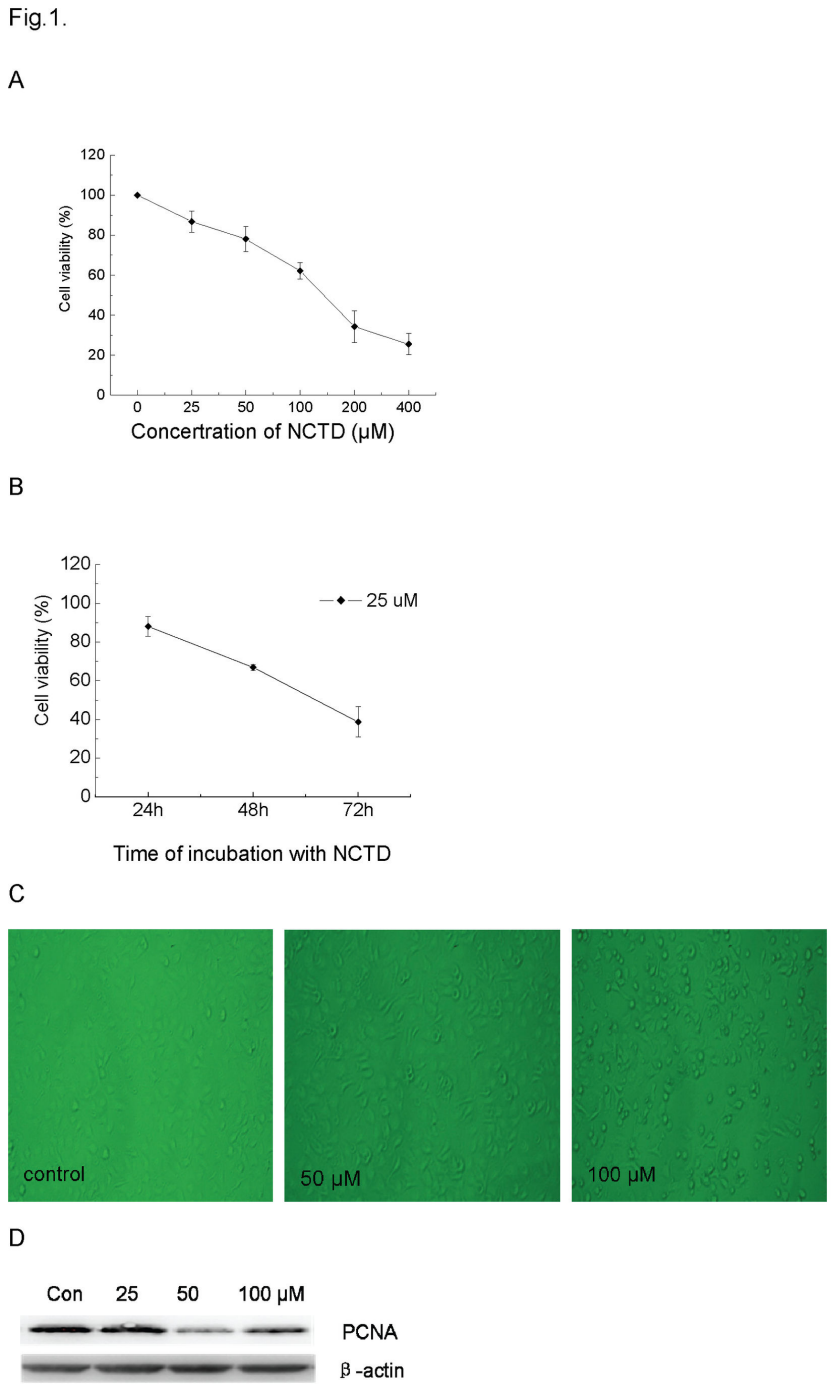
Growth inhibition and the morphological alteration of DU145 cells exposured to different concentrations of NCTD for 24 h. Plot A, cell viability of DU145 treated with indicated concentrations of NCTD (0-400 μM) for 24h. Plot B, cell viability of DU145 treated with 25 μM NCTD for 24, 48, and 72 h, respectively. Plot C, morphological alterations of DU145 cells exposed to different concentrations of NCTD. Plot D: protein immunoblots were probed with PCNA antibody under western blot assay.

As shown in Figure 1A, when DU145 cells were exposed to NCTD with various concentrations from 25 to 400 μM for 24h, the cell proliferation was inhibited in a dose-/time- dependent manner. When DU145 cells were incubated with 25 μM NCTD for 24 h, 48 h and 72 h, the corresponding proliferation inhibition rates were increased to 12.91 %, 33.14 % and 61.26%, respectively (Figure 1B). The cell morphology was changed after the treatment with NCTD for 24h (Figure 1C). The normal DU145 cells were polygonal in appearance with well-defined intercellular spaces. After the treatment with 50 μM NCTD, a significant number of cells became rounded-up. 100 μM NCTD could result in cell anoikis so that many cells were detached from the culture plate. In addition, the western blotting assay showed that 50 and 100 μM NCTD could suppress the expression of PCNA, a well-known marker of proliferation (Figure 1D).

### NCTD induced cell death relied on mitochondrial dysfunction

**Figure 2:**
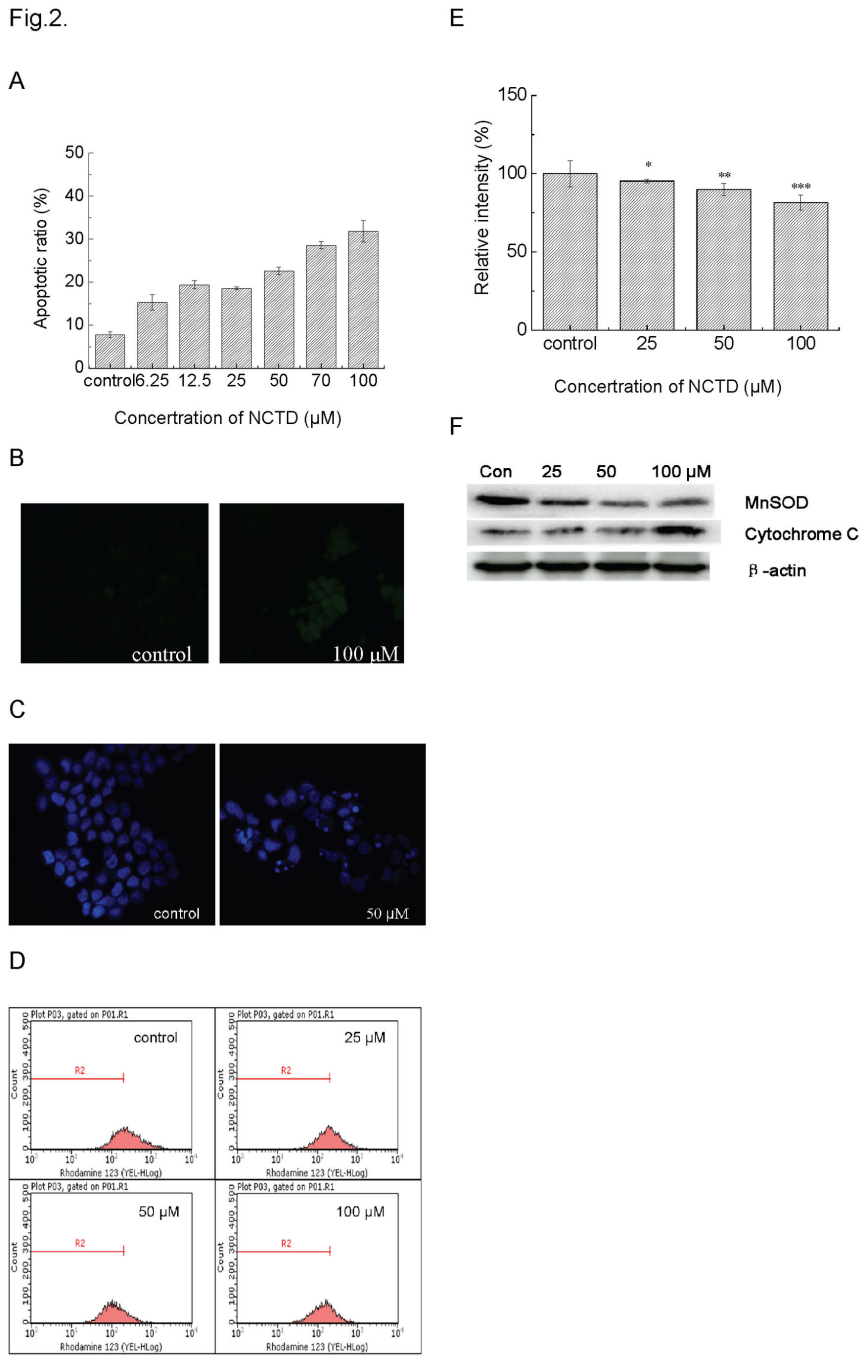
Apoptosis induction in DU145 cells exposed to0, 6.25, 12.5, 25, 50, 70, or 100 μM NCTD for 24 h. Plot A, apoptotic cells were measured with cytometry using a FITC-Annexin V Apoptosis Detection Kit. Plot B, the typical images of apoptotic cell nuclei was scored after treatment with 50 μM NCTD for 24 h (×40, Hoechst 33258 staining). Plot C, apoptotic cells detected with TUNEL staining assay. Plot D, cells were stained with Rhodamine 123 and mitochondrial membrane potential was analyzed by flow cytometry. Plot E, the fluorescence intensity were calculated to represent the disruption of mitochondrial membrane potential. *P <0.05, **P <0.01, ***P<0.001 vs. Control. Plot F: Immunoblots were probed with MnSOD and cytochrome *c* primary antibody.

The flow cytometery assay revealed that the population of Annexin-V-positive and PI-negative cells in the NCTD-treated population increased along with the concentration of NCTD so that the apoptotic ratio increased from 7.54 ± 0.67 % control) to 27.3 ± 2.47 % (100 μM) (Figure 2A). Since the mitochondria depolarization was an irreversible change during apoptosis process, we measured the change of mitochondrial membrane potential (MMP). The histogram of flow cytometry assay illustrated that the peak of rhodamine 123 had a left-shift along with the increasing NCTD concentrations, indicating that NCTD could induce depolarization of the inner mitochondrial membrane in a dose-dependant manner (Figure 2D). The percentage of the cells with mitochondria depolarization was 4.8 %, 10.13 % and 18.52 % corresponding to were 25, 50 to 100 μM of NCTD, respectively (Figure 2E). 

On the other hand, cytochrome *c*, a key initiator in the mitochondrial apoptosis pathway, can be released from the inter-membrane of mitochondria after mitochondria depolarization. A representative result of cytochrome *c* release monitored by immunoblotting analysis after 24 h of NCTD treatment was shown in Figure 2F. As expected, the generation of cytochrome *c* was induced by NCTD in a dose-dependent manner suggesting that NCTD could induce apoptosis through an intrinsic mitochondrial pathway. Meanwhile, MnSOD, as another self-defended antioxidase molecule that was located in the matrix of mitochondria, was found decreased along with the increasing concentrations of NCTD. 

### ROS-mediated DNA damage was related to cell death induced by NCTD

**Figure 3:**
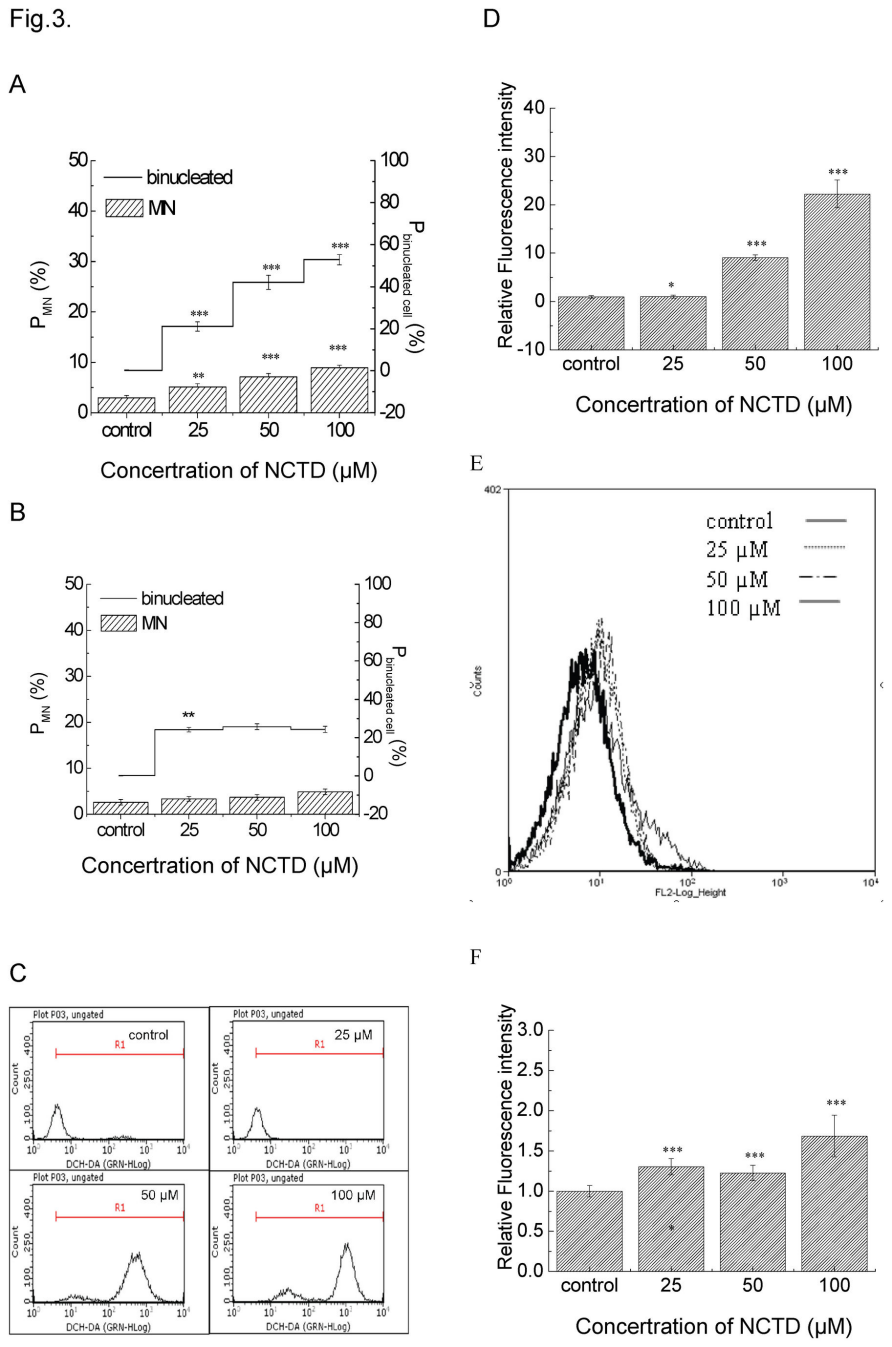
NCTD induced DNA damage and intracellular ROS level. Plot A, the percentage of cells with MN and the percentage of binucleated cells after NCTD exposure for 24 h. Plot B, NCTD-induced MN was decreased by extrogenerous SOD-PEG. Plot C, DCH-DA fluorescence intensity was measured at 24 h by flow cytometry. Plot D, The values of fluorescence intensity were calculated in different concentration groups. *P <0.05, **P <0.01, ***P <0.001 vs. Control. Plot E, mitochondrial ROS were measured with mitoSox probe. Plot F, the relative fluorescence intensity of MitoSox in the NCTD-treated cells. ***P <0.001 vs. Control.

MN, which generated from chromosome fragments at the anaphase during nuclear division, can be used as a biomarker of DNA damage [21]. After NCTD treatment, the percentage of cells with MN increased from 2.96±0.46 % (control) to 5.13±0.61 %, 7.13±0.64 % and 8.93±0.50 % (corresponding to 25, 50, and 100 μM NCTD, respectively) (Figure 3A). In addition, the ratio of binucleated cell ascended with the increasing concentrations of NCTD and almost half of cells possessed binuclei treated with 100 μM NCTD. These phenomena were inhibited when 500U/ml SOD-PEG was added into the culture medium 1 hour prior to NCTD so that the percentage of cells with MN and binucleated cells were decreased significantly, especially in the high-concentration NCTD groups (shown in Figure 3B). 

Oxidative stress is a crucial incentive factor for DNA damage. In order to determine whether NCTD-induced DNA damage was relevant to oxidative stress, the intracellular ROS level was measured using the fluorescence probe (DCFH-DA). Corresponding results demonstrated that NCTD could enhance the ROS level while 50 μM NCTD dramatically increased the intracellular ROS level to 22-fold relative to the control (Figure 3C and D). As mitochondria play fundamental role in oxidative defense, under various pathological conditions, mitochondria may emerge as a significant site of the generations of superoxide and other reactive oxygen and nitrogen species. In our experiments, mitochondrial superoxide generation was measured with MitoSOX Red. The result showed that NCTD induced a dose-dependent increase in the intensity of MitoSOX fluorescence. 

### ATP was depleted in NCTD-treated cells

**Figure 4:**
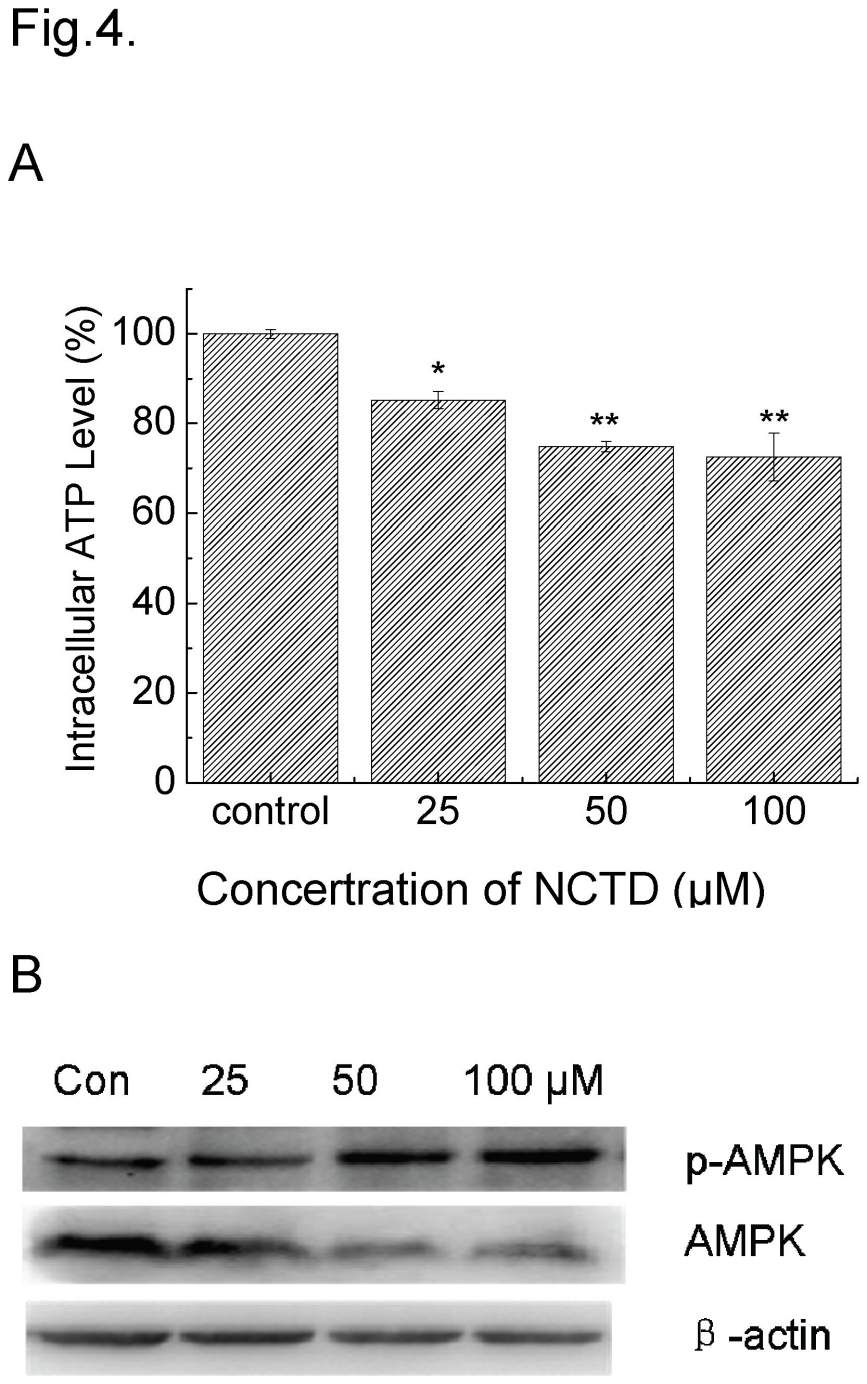
The ATP Levels in DU145 cells exposed with NCTD for 24 h. Plot A: the related light unit (RLU) was used to describe the activity of luciferase. The values were normalized with the number of cells. *P <0.05, **P <0.01 vs. Control. Plot C and B: immunoblots were probed with indicated antibodies. Lysates were from the cells exposed to 25, 50, and 100 μM NCTD for 24 h.

We observed a progressive lowering of ATP in the cells after treated with (25 μM to 100 μM) NCTD, which may be attributed to the reason that ATP was consumed a lot in the process of the DNA damage repaired (Figure 4A). As a sensitive sensor of intercellular ATP, the activation of AMPK was consistent with the level of intercellular ATP (Figure 4B). The depletion of ATP did not induce the up-regulation of the expression of total AMPK but activated the phosphorylation of AMPK molecular. 

## Discussion

To date, chemotherapy still remains one of the most frequent and effective treatments for malignant tumors. Cell proliferation inhibition and apoptosis induction are the critical indexes for evaluating the therapeutic effect of an anticancer drug. Previous studies have demonstrated that NCTD caused proliferation inhibition in several tumor cell lines *in vitro* and *in vivo* [12,14,22,23]. Our results indicated that NCTD could decrease the viability of DU145 in a dose-/time- dependent manner, and apoptosis was the major pattern of cell death associated with morphological and biochemical changes. 

The mitochondrion is the most important sensor for apoptosis [24]. The stimuli factor could damage the integrity of the mitochondrial membrane and led to the collapse of MMP by increasing the permeability of cell membrane [17]. When the above happened, oxidation of metabolites concomitant with electron flux is uncoupled to proton pumping, resulting in the dissipation of the trans-membrane proton gradient. As a consequence, electrons could escape from the electron transport chain of mitochondria and contribute to the production of ROS [25]. When the generation of ROS is not overwhelmed by antioxidant protein, ROS accumulation in the mitochondria could cause the depolarization of mitochondrial membrane by lipid peroxidation [26]. Manganese superoxide dismutase (MnSOD), an intrinsic ROS scavenger of mitochondria, was localized in the matrix of mitochondria. The decrease of MnSOD not only impairs the cell self-defense against ROS, but also damages mitochondrial membrane. As a result, permeability transition pore (PTP) could be formed in mitochondrial so that cytochrome *c* was released from mitochondria to cytosol, which would trigger a series of downstream target gene expressions and cell apoptotic pathways [27]. It has been revealed that NCTD caused accumulation of cytosolic cytochrome *c* and activation of caspase-9 but not Fas-FasL pathway in a variety of cancer cells, for example, human oral cancer cells, melanoma cells and leukemic cells [28–30]. Accordingly, the NCTD-induced cell apoptosis is relative to the ROS- mediated mitochondrial damage. 

DNA damage induced by oxidative stress also contributes to the proliferation depression of NCTD-treated cells. This study showed that NCTD yielded a high percentage of cells with MN, in consistent with other studies where DNA strand break were induced by NCTD [28,31]. Our data showed that the intracellular level of ROS rose with the increasing concentrations of NCTD. Under the physical condition, low level of ROS acts as the second messenger for the regulation of proliferation and differentiation [32]. Once the balance between the generation of ROS and the activity of antioxidant proteins was disturbed, cells would suffer from the oxidative damage and lipid peroxidation [33]. It was shown that 10 mM NAC attenuated the increase of NCTD-induced ROS generation and anti-proliferation in HepG_2_ cells [34]. Consistently, our results showed that exogenous SOD-PEG associated with NCTD treatment eliminated the generation of MN in DU145 cells. A recent research also indicated that H2AX, a well-known specific and sensitive reporter of DNA damage could up-regulate the activity of NADP(H) oxidase (Nox), thus increasing the generation of ROS and aggravating DNA damage [35]. It is well-known that H2AX could be phosphorylated and recruited around the site of chromatin domains flanking double-strand breaks to activate the repair pathways [36]. This mechanism might be involved in NCTD-induced DNA damage and oxidative stress. 

On the other hand, due to the accumulation of DNA damage, a higher proportion of binucleated cells were also observed in the NCTD-treated cells, indicating the delay of cell mitosis. Usually, the accumulation of DNA damage can activate cell cycle checkpoint and slow the cell cycle progression in order to repair DNA damage, which partly contributes to the cell proliferation depression. These results were consistent with the effects of NCTD on leukemia cells and glioblastoma cells [37–39]. Besides, the inhibitory of NCTD on PP2A activity also take part in the retard on cell cycle progress by regulating cyclin D1, D3, E, A, B and so on [40]. 

The depletion of ATP induced by NCTD not only resulted from the oxidative damage, but also contributed to the inhibition of cell proliferation by influencing cell metabolism and enhancing the oxidative stress. It is well-known that ATP is the main energy source for the synthesis and transport of macromolecules and proteins, including DNA and RNA. Some studies showed that ATP was necessary for cell proliferation to act as a substrate in signal transduction processes. Vascular endothelial growth factor isoform VEGF-A_165_ is a primarily endothelial cell-specific mitogen. Its complex of binding to ATP was essential for inducing proliferation of human umbilical vein endothelial cells, rather than itself [41]. Under the pathological conditions, tumor necrosis factor alpha activated and promoted the proliferation of glial cell which was associated with the activation of P2X_7_ receptor by ATP [42]. We found that ATP was used up after DU145 cells treated with NCTD, which revealed a possible mechanism involved in the down regulation of the proliferation-relate genes PCNA, Ki-67 and p27 in gallbladder carcinoma cells [43]. It was verified in our study that the expression of PCNA was decreased by NCTD in DU145 cells. Concurrently, the metabolic processes, which leads to repair or fixation of DNA damage, is greatly influenced by a continuous supply of metabolic energy. It has been demonstrated that ATP was necessary for the rejoining of DNA breaks in repairing the potentially lethal damage [44]. When DU145 cells were treated with NCTD, cells consumed a large amount of ATP to fix the damage and enhanced the severe depletion of intercellular ATP. It was speculated that cells would compensate and increase mitochondrial oxidation which is the major source of ATP generation to improve their bioenergetics to rescue the normal physical metabolism. What’s worse, ROS is the by-product in oxidative phosphorylation process. Furthermore, when nutrients wane, cells might become more vulnerable to apoptosis. Some important effectors communicated signals between metabolism and apoptosis [45]. AMPK was one of the sensors, which activated by the increase of the ratio of AMP to ATP and impeded cell growth and proliferation by inactivating the synthesis of fatty acids and cholesterol [46]. Activation of JNK-p53 signal axis and inhibition of mTOR activity were involved in AMPK-induced growth inhibition [47]. 

In conclusion, our observations highlighted that apoptosis is the major form of cell death, as well as the therapeutic effects of NCTD on inhibition the proliferation of NCTD accompanied with MMP collapse and mitochondria dysfunction. DNA damage and depleting ATP pool induced by the accumulation of intercellular and mitochondria ROS triggered the molecular pathway.
